# Climate change and mental health: the rising tide of eco-distress

**DOI:** 10.1177/17579139251333289

**Published:** 2025-07-22

**Authors:** Bill Sheate

**Affiliations:** Bloomsbury Therapy Centre, Bristol House, 80a Southampton Row, London WC1B 4BA, UK; Emeritus Reader, Centre for Environmental Policy, Imperial College London, London, UK

## ECO-Anxiety/ECO-Distress

‘Eco-*anxiety*’ is: *‘. . . a chronic fear of environmental doom’*.^1^ Generally associated with the climate crisis, it also relates to other environmental concerns from pollution to biodiversity loss. It is not a psychological disorder, but a specific contextual manifestation of a range of negative emotions. Eco- (or climate-) anxiety has become an umbrella term for all the other eco-emotions, including anger and sadness, but a more encompassing term perhaps is ‘eco-*distress*’^i^ which covers most of the negative emotions associated with environmental and climate change.^2,3^

‘Grief’ features strongly in the academic literature on eco-emotions, most relevant to those directly affected by extreme weather events. For many, though, their experience is usually less immediate, more anticipatory, and therefore often features a strong component of fear/anxiety about the future and anger at humanity’s apparent inability to act. While some might talk of a sense of loss, this can be as they struggle with their thoughts and feelings, for example, around hopelessness and helplessness. The nature and taxonomy of psychosocial responses to environmental change is open to extensive *academic* exploration,^4,5^ although sometimes this is of limited value to therapeutic *practice*.

At its most severe, eco-distress typically involves safety-seeking behaviour and multiple underlying maladaptive processes: over-thinking (worry, rumination), intolerance of uncertainty, avoidance, procrastination, stuck attention, social isolation, and doom scrolling (so readily facilitated by social media that it can occupy hours of each day). All contribute to, maintain, and exacerbate high levels of anxiety, sadness, or anger and can lead to paralysis in day-to-day decision-making.

## The Evidence for ECO-Distress

Eco-distress is widespread, often among young people but also more generally.^6,7^ In 2018, 51% of respondents in the US listed climate change as ‘a somewhat or significant source of stress’.^8^ In a more recent survey of 10,000 young people (aged 16–25) from 10 countries worldwide, 59% were very or extremely worried, with 84% at least moderately worried. Furthermore, >50% reported being sad, anxious, angry, powerless, helpless, and guilty, and >45% said their feelings about climate change negatively affected their daily life and functioning.^6^ Not surprisingly, those vulnerable to anxiety or depression appear more vulnerable to eco-distress.^9^

## The Consequences of ECO-Distress

Eco-distress often presents as a ‘stuck’ focus of attention, where thoughts are dominated by the past or the future, where experiential avoidance is prevalent along with fusion about the meaning of negative automatic thoughts, with a range of cognitive distortions. The environment often becomes either the dominant value or pervades all other personal values to the extent that it becomes paralysing, for example, struggling with impossible choices for low-carbon living when shopping for food. The eco-distressed may find they are unable to actively engage with other values (relationships, friends, family, work, etc.). Ironically, through avoidant behaviour, they may even stop engaging with the very thing they value most highly – nature and the environment.

**Figure:**
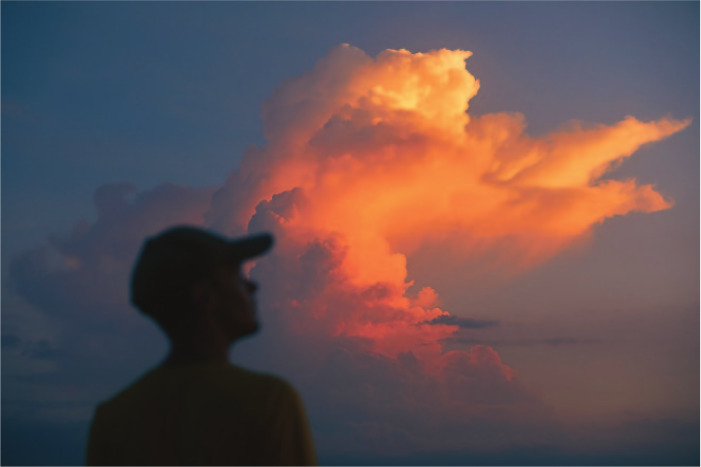


**Figure 1:**
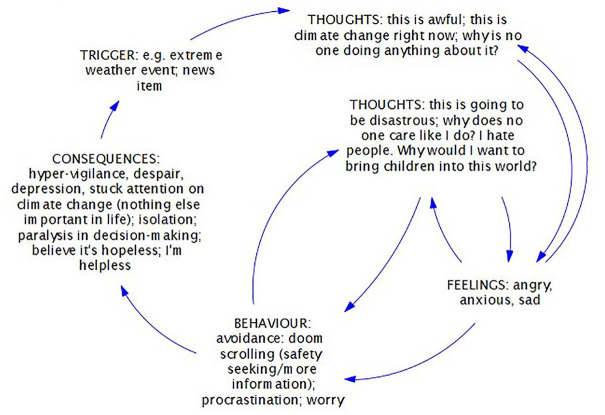
Generalised, illustrative vicious cycle for severe eco-distress

The dominance of future thinking, and fear of the future, while not an unnatural response to climate change, is not helpful if it results in paralysing levels of worry, to the extent that the eco-distressed often get anxious about being anxious (which feeds into experiential avoidance as a coping strategy) (see Figure 1).

## Why and How ECO-Distress Might Impact Mental Health in the Future

Vulnerable communities worldwide are already affected disproportionately by climate change, through increasing frequency and extremes of weather events (floods, drought, fires, heatwaves) and therefore also by impacts on mental health, through loss of life, livelihoods, property and migration.^9,10^ For others, such events may lead to an increasing sense of hopelessness and helplessness among those already prone to anxiety or depression, and these stresses are also likely to manifest themselves in the wider population as impacts are experienced more directly.

In seeking to ‘treat’ eco-distress, the aim is not to eradicate the emotional response, rather, it is to shift or gain perspective, accepting that these emotions are normal human responses to an existential threat, but that in extreme forms, they are maladaptive. A common desire is ‘to find joy in life again’ and that often involves (re)connecting to one’s personal values.

Pro-environmental behaviour – individual and collective – to further climate change action can offer a helpful channelling of emotions,^11,12^ although may risk exacerbating the focus of attention on the single issue and their emotional struggle. Actions that are inconsistent with other personal values, for example, confrontational demonstrations/campaigns, can cause a spiral of despair and depression. But behaviour change can contribute to wider action in service of multiple values (e.g. being more present, connecting with friends, community, nature), such as through volunteering or practical action that makes a tangible difference on the ground.^13^

## The Lessons for Public Health Professionals

Many of our existing therapeutic models offer effective ways to shift perspective from worrying about the future to greater present moment awareness, a reconnectedness with nature, people, and their surroundings, and towards action in the present where individuals have agency, including in relation to other things that are important to them.

There will be an increasing need for public mental health preventive action, including education, mental health promotion, and protection, especially for children and young people, community-based strategies, green prescribing, and resilience skills training that equip people to better process their climate-related emotions and build self-efficacy.^10,11^ Some therapists and other mental health professionals, however, may not feel confident in their own environmental knowledge and understanding, and the eco-distressed can find it exhausting having to explain environmental issues to others. Consequently, additional resourcing and training for health professionals in these areas need urgent consideration by professional bodies.

How climate change is communicated and how eco-distress is framed into the future may influence the perception of public health threats and solutions;^14^ greater awareness of eco-distress could, conceivably, contribute to an unhelpful narrative that the situation is hopeless. Care will be needed in public health messaging, and in conducting research, to ensure maladaptive eco-distress is not further magnified, while the continuing impact of eco-distress on wider public mental health further strengthens the need for visible and effective action on climate change.

## Notes

i. Collins Dictionary definition of distress: ‘great pain, anxiety or sorrow’.
